# The place of advanced machine learning techniques in building pancreatic adenocarcinoma survival and recurrence prognosis models

**DOI:** 10.3389/fonc.2025.1727806

**Published:** 2025-12-17

**Authors:** Mihaela-Flavia Avram, Daniela-Cornelia Lazăr, Mihaela-Ioana Mariş, Alexandru-Ştefan Cucui-Cozma, Marius-Sorin Murariu

**Affiliations:** 1 Department of Surgery X, 1st Surgery Discipline, “Victor Babeş” University of Medicine and Pharmacy Timişoara, Timişoara, Romania; 2 Abdominal Surgery and Phlebology Research Center, “Victor Babes” University of Medicine and Pharmacy Timisoara, Timisoara, Romania; 3 Department V of Internal Medicine I, Discipline of Internal Medicine IV, “Victor Babeş” University of Medicine and Pharmacy Timişoara, Timişoara, Romania; 4 Department of Functional Sciences, Pathophysiology, “Victor Babes” University of Medicine and Pharmacy Timisoara, Timişoara, Romania; 5 Center for Translational Research and Systems Medicine, “Victor Babes” University of Medicine and Pharmacy Timisoara, Timisoara, Romania

**Keywords:** artificial intelligence, pancreatic adenocarcinoma, advanced machine learning, cancer survival prediction, cancer recurrence prediction, machine learning cancer prediction

## Abstract

**Background:**

Pancreatic ductal adenocarcinoma (PDAC) is a highly lethal malignancy, and traditional prognostic methods, such as TNM staging, often fail to accurately predict outcomes. This review evaluates the use of machine learning (ML) to improve PDAC prognosis.

**Methods:**

A systematic literature search of PubMed and Google Scholar was conducted, identifying 12 studies that applied ML algorithms to predict survival, recurrence, and metastasis in patients with PDAC.

**Results:**

Various algorithms, including Random Forests, XGBoost, and Deep Learning, demonstrated superior predictive performance compared to the TNM staging. Models using multimodal data—combining clinical, radiomic, and genomic features-yielded the highest accuracy for predicting overall survival and early liver metastasis.

**Conclusion:**

ML offers a significant advantage in analyzing complex medical data to refine risk stratification and support personalized PDAC treatment. However, current models are limited by their small datasets and retrospective designs. Future research requires prospective validation to translate these ML tools into clinical practice.

## Introduction

Pancreatic ductal adenocarcinoma (PDAC) is one of the most resistant and fatal cancers, ranking as the seventh highest cause of cancer-related mortality globally (1). The incidence and mortality of PDAC are still rising, and the disease is expected to have a poor prognosis and a low resection rate despite major advancements in detection and treatment (2). PDAC, because of its retroperitoneal anatomical positioning and subtle early manifestations, leads to approximately 80%–85% of patients presenting at an advanced stage at the time of diagnosis (3).

Various criteria can be used to predict PDAC prognosis. Systemic immunological inflammation in cancer patients is strongly linked to metastasis and poor prognosis, according to a growing body of research (4–7). The most prevalent tumor marker for PDAC in clinical practice is carbohydrate antigen 19 9 (CA19-9), however its low sensitivity and specificity still limit its use in prognostic prediction (7–9). To predict the recurrence and survival of patients with PDAC, TNM stage and pathological grade are also used (10). There have also been reports of a correlation between the overall survival of patients with PDAC and serum indices such as albumin, serum bilirubin, and serum alkaline phosphatase (11). Nonetheless, the correlation between variables and the prognosis of patients is still controversial and requires further additional investigation.

In medicine, the use of AI and machine learning mostly helps with outcome prediction, risk assessment, and disease diagnosis. Researchers have examined diseases that require early diagnosis, including those of the skin, liver, heart, and Alzheimer’s disease, using various AI-based machine learning models (12). Thus, machine learning could improve the prognosis and outcome prediction of pancreatic ductal adenocarcinoma.

In this review, we investigated the potential of machine learning models in pancreatic adenocarcinoma prognosis, with an emphasis on the outcomes and the use of advanced machine learning techniques in the prediction of survival and recurrence.

This review is structured as follows:

- Introduction—contains information about PCAD and its prognosis and the use of ML in survival prediction, the purpose of the paper and its content.
- Material and methods—describing the selection process of included studies.
- Results—a summary of the selected studies organized in subsections according to their aim: survival (after surgery and overall), recurrence, and liver metastasis prediction. Data selection, feature extraction, ML algorithms used, and performance evaluation are included.
- Discussion—it highlights the findings, compares them to other prognostic methods and highlights their advantages.
- Challenges and future directions—assessing the existing issues and prospective development possibilities.
- Conclusions—summarizes the key findings and their implications.

## Materials and methods

We conducted a comprehensive literature review to identify studies describing pancreatic adenocarcinoma prognostic models based on machine-learning algorithms. PubMed and Google Scholar were systematically searched using the selected keywords and terms. These included “artificial intelligence,” “pancreatic cancer,” “prognosis,” and “machine learning.” Multiple combinations of these terms were applied to each database using appropriate Boolean operators (OR/AND).

All studies on the use of ML for PDAC prognosis were included in this review. The following were excluded: (1) Other types of studies than original (meta-analyses, systematic reviews, narrative reviews, comments, and case reports); (2) Articles lacking full text in English; (3) Studies that did not use ML algorithms; and 4. Studies that did not address PCAD prognosis.

The literature search retrieved 1,017 articles, from two databases (PubMed and Google Scholar). A total of 620 abstracts were screened, and 46 full-text articles were assessed for eligibility. Studies in which no prognostic models were developed, AI was not used to develop the model, and future perspective articles were excluded. Finally, 12 studies were included (**Table 1**). The flow diagram is shown in **Supplementary Figure 1** (25).

**Table 1:** Overview of the included studies.

Prognostic area	Author	Year	n	Algorithms used	AUC/C-index	Best performing model	Variables used	Obs.	Potential use
prediction of long-term survival for borderline resectable pancreatic cancer with upfront surgery	Huang et al. (13)	2025	104	logistic regression SVM Random forests decision tree XGBoost	0.864	logistic regression	age vascular invasion length vascular morphological malformation local lymphadenopathy	Retrospective study Lacks external validation Small number of patients	deciding the best treatment option
1 year survival after pancreaticoduodenectomy	Ahmed et al. (14)	2025	16772	gradient boosting model logistic regression random forest decision tree	0.689	gradient boosting	age tumor size differentiation neoadjuvant therapy	Retrospective study Lacks external validation Weak ability to differentiate	Provide a decision tool for choosing the best treatment to optimize survival
predicting 1-, 3-, 5-year survival	Hu et al. (15)	2024	142	Pretrained DL neuronal networks for pathologic images Clinical model Combined model	0.77	combined model	H&E image 9 laboratory tests, age, sex, tumor site, vascular invasion, TNM classification, tumor grade Combination of the two models	Retrospective study Lacks external validation	integrating clinical and pathological data for overall survival prediction for personalized treatment strategies
survival prediction modeling	Xiao et al. (16)	2025	117	COX regression DL -DeepSurv	0.724	DeepSurv model	BCAT-1 AMY CA-12-5	Part of a complex study which included diagnosis and treatment personalized recommendations Retrospective study Lacks external validation Small number of patients	elaborate personalized treatment recommendations to provide further survival benefits
long-term *vs* short-term survival	Schuurmans et al. (17)	2025	762	combined multimodal AI model clinical model-two-layer neural network imaging model-EfficientNet-B4	AUC 0.675	multimodal AI model	CT images age, CA19-9, CEA, BMI, unexpected weight loss, ECOG performance status	Retrospective study Strong external validation	Prognostic tool in treatment decision making
3-year survival prediction differentiated on sex	Ojha et al. (18)	2025	107	Random Forest, AdaBoost Logistic Regression Decision Tree Linear Support Vector Machine	0.67(M)/0.83(F)	Random forests	gene expression profiles	Retrospective study Web application available	Improved Survival prediction based on genomic profile and sex
1,3,5- year survival prediction	Luo et al. (19)	2025	250 + 61-external validation	162 models-combining:RSF, Enet, StepCox, CoxBoost, plsRcox, superpc, gbm, survival-SVM, Ridge, obliqueRSF, xgboost, CForest, and CTree	1 y–0.77 3 y–0.79 5–0.76	Random Survival Forests	innate immune barrier–related gene	Retrospective study	Stratification of patients into treatment groups Advanced precision oncology
survival- dividing patients into low or high-risk groups	Ge et al. (20)	2025	168	RSF survival-SVM Ridge Regression Elastic Net LASSO SuperPC GBM plsRcox CoxBoost Stepwise Cox Regression	0.682	Ridge regression	transcriptomic, methylation, and mutational data	Retrospective study	Personalized therapeutic strategy
overall survival and mutational status (KRAS, TP53)	Zaccaria et al. (21)	2025	278 patients 598 differentially methylated CpG sites	Random forests	0.79 (for KRAs) 0.77, (TP53) -0.77 (KRAS/TP53 co-mutation)	Random forest	differentially methylated CpG sites	Retrospective study	Risk classification
1,3,5- year survival prediction	Wang et al. (22)	2025	1034 samples	117 algorithmic (StepCox, GBM, RSF, CoxBoost, Enet, plsRcox, survival-SVM, SuperPC, Lasso, Ridge regression)	1-year = 0.795, 3-year=0.776 5-year=0.761	StepCox[both] and Ridge	programmed cell death-related genes	Retrospective study	Prognostic signature for personalized management strategies
preoperative prediction for early recurrence	Yan et al. (23)	2025	177 patients	logistic regression	0.712	logistic regression	radiomics, CA19–9 and visceral to subcutaneous fat volume ratio	Retrospective study No other alternative ML algorithms tested	Guiding tool for personalized treatment
early liver metastasis prediction	Zhu et al. (24)	2025	538	Logistic Regression K-Nearest Neighbors Random Forest XGBoost Decision Tree Gradient Boosting Machine Support Vector Machine	0.901	XG Boost	BMI, fatty liver, Child-Pugh score, tumor location, N stage, differentiation, tumor emboli, nerve invasion, adjuvant chemotherapy	Retrospective study Online model available	Improving personized treatment decision

## Results

**Figure 1:**
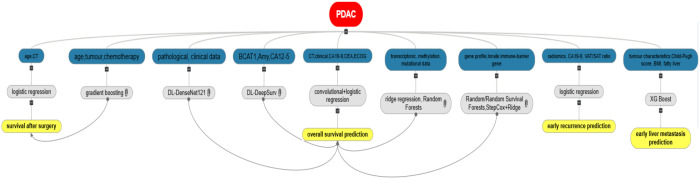
Overall view of the studies, data used, best performing algorithms, and outcome.

The studies included had three major predictive objectives: survival, recurrence, and metastasis. **Figure 1** summarizes these studies.

### Survival prediction

#### Survival after surgery prediction

To predict long-term survival (2 years) in patients with borderline resectable pancreatic cancer undergoing upfront surgery, Huang et al. used four variables: age, vascular invasion length, vascular morphological malformation, and local lymphadenopathy. Logistic regression along with support vector machines (SVM), random forest, decision tree, and XGBoost were tested. The AUC stratified cross-validation in the logistic regression (0.864) was better than SVM (0.693), random forest (0.789), decision tree (0.790), and XGBoost (0.726) (13). Their findings may contribute to the selection of a personalized treatment option for these patients.

As curative surgery, including pancreaticoduodenectomy, does not always provide the expected results in terms of survival, with a significant percentage of patients not reaching the 1-year survival point, Ahmed et al. used machine learning algorithms to predict the odds of futile surgery. They utilized preoperative input variables (age, tumor size, and poor differentiation as key predictors of fatal outcomes, while neoadjuvant therapy was associated with better survival) to develop a predictive model. The algorithms employed were: gradient boosting model (showed the best performance with an AUC of 0.689), logistic regression (0.679), random forests (0.675), and decision tree (0.664) (14). Although the performance of the model is modest, it provides a good starting point for future research.

More complex algorithms are not necessarily superior. In predicting survival for borderline resectable patients, simple Logistic Regression outperformed advanced models such as SVM, Random Forest, and XGBoost. ML is used to identify patients for whom surgery will not provide a survival benefit (death <1 year). The key predictors of fatal outcomes are age, tumor size, and poor differentiation.

#### Overall survival prediction

Hu et al. created a complex prognostic model created by using a pathological risk model combined with a clinical risk model was constructed. For the pathological model, pretrained deep neural network models (MobileNet_v3_small, ResNet18, and DenseNet121) were used to extract features from representative hematoxylin and eosin (H&E)-stained digital slides/patients. The DenseNet121 model performed best, with a C-index of 0.73 in the external validation. For the clinical model, nine preoperative laboratory tests, sex, age, tumor site, tumor differentiation, and TNM classification were included. The combined model, using the 0.42 pathological model plus the 0.26 clinical model, showed a performance of 0.77 for the testing set, showing an improvement compared to the performance of the single models as well as the survival prediction made by only utilizing TNM classification (15).

Xiao et al., in a complex study using data from 117 patients with PDAC, extracted three features: Branched-chain Amino Acid Transaminase 1(BCAT1), amylase (AMY), and carbohydrate antigen 12-5 (CA12-5). The data were used to create a COX proportional hazards model and a DeepSurv model (a deep learning survival analysis model based on the Faraggi–Simon network model) for survival prediction. The DL model outperformed the COX model, with a C-index of 0.724 for the validation set. The authors used this model to further elaborate on personalized treatment recommendations to provide survival benefits (16).

Another promising study on PDAC prognostication using DL models was conducted by Schuurmans et al. They created two AI models. The first was a clinical model developed as a two-layer neural network using data retrospectively collected from 401 patients with PDAC. The variables used were age, CA19-9, CEA, BMI, unexpected weight loss, and ECOG performance status. The prediction of long- *vs* short-term survival reached an AUC of 0.638 in the external validation. An imaging model based on CT images from the same patients, created using a pure convolutional model (EfficientNet-B4), provided an AUC of 0.55 on external validation. The best predictive power was for the combined multimodal model, which consisted of a logistic regression using the risk scores provided by the previous two models. The performance on external validation reached an AUROC of 0.675 (17). This study is one of the few that provides data on the external validation of prognostic values. Although the discrimination power is not ideal, it shows an improvement compared to traditional PDAC prognostication using TNM staging.

A different approach to building ML prognosis models for PDAC was used with RNA-sequencing data. Ojha et al. created a 3-year prognosis model using gene expression data. By testing different ML techniques, the best-performing algorithm was determined to be random forests. To provide additional prognostic improvement, the model was individualized for male and female patients, thus providing supplementary personalization and an increase in the AUC value during external validation (18). This study illustrates the utility of ML in handling complex datasets and creating robust predictive models for PDAC.

Luo et al. employed an ML algorithm to construct a survival model based on innate immune cell barrier genes for pancreatic cancer, selected from a set of 1,356 unique genes associated with monocytes/macrophages, dendritic cells, NK cells, and neutrophils extracted from multiple databases. To select the best-performing algorithm, 162 models were constructed based on a combination of the following: RandomSurvivalForests, Enet, StepCox, CoxBoost, PartialLeastSquareRcox, supervisedPrincipalComponents, Gradient Boosting, survival-SVM, Ridge, obliqueRandomSurvivalForest, xgboost, CForest, and CTree. On external validation, the model based on the Random Survival Forest algorithm showed the best performance when constructed using 84 cancer diagnosis risk genes (CDRG) with a c-index-0.615. This model proved to have a good prognostic value for predicting survival at 1-, 3-, and 5-year (AUC—0.77, 0.79, 5–0.76) (19).

By integrating transcriptomic, methylation, and mutational data from 168 pancreatic cancer samples, Ge et al. identified molecular subtypes and built a pancreatic cancer prognostic model. The researchers used ten machine learning algorithms—random survival forest, survival-SVM, ridge regression, LASSO, elastic net, SuperPC, GBM, plsRcox, CoxBoost, and stepwise Cox—forming 101 model combinations. The final prognostic signature was built using ridge regression, which performed best (20). It divides patients into low- and high-risk groups, with high risk indicating worse survival outcomes. This study established a robust multi-omics-based classification of pancreatic cancer and provided a foundation for personalized therapeutic strategies.

Using a random forest algorithm trained on 598 differentially methylated CPG sites extracted from a publicly available dataset, along with clinical features and top mutated genes, Zaccaria et al. developed an ML model that has a good ability to predict KRAS, TP53, and KRAS and TP53 co-mutations on external validation. Furthermore, they used consensus clustering of methylation profiles to create four subgroups with different survival characteristics. An eXtreme Gradient Boosting classifier was used to identify epigenomic prognostic determinants. They successfully used a combination of several ML algorithms to integrate diverse and voluminous data to create a prognostic model for PDAC (21).

Another PDAC prognostic model was developed by Wang et al. using transcriptomic data from 1,034 samples, focusing on programmed cell death-related genes. The authors selected 17 genes, which were further reduced to a six-gene signature, to build a prognostic model. A total of 117 algorithmic combinations were generated using a combination of ML algorithms (StepCox, GBM, RSF, CoxBoost, Enet, plsRcox, survival-SVM, SuperPC, Lasso, and Ridge regression). The best performing model proved to be the one combining StepCox[both] and Ridge, providing an AUC for prediction of survival at 1-year = 0.795, 3-year = 0.776. and 5-year = 0.761 on external retrospective validation (22).

In summary, combining different data types yields better predictions than using a single data source. Deep Learning models extracting features from H&E slides (DenseNet121) combined with clinical data outperformed TNM staging. Combining CT imaging features (Radiomics/Deep Learning) with clinical markers (CA19-9) improves risk stratification.

In studies using specific biomarkers (e.g., BCAT1, Amylase), Deep Learning models (DeepSurv) demonstrated superior performance (C-index 0.724) compared to traditional Cox proportional hazards models. ML algorithms (specifically Random Forests and Ridge Regression) excel at processing complex genomic data (RNA-Seq, methylation, and immune genes) to build prognostic signatures. The creation of separate models for male and female patients improved the prognostic accuracy.

### Predicting recurrence

To predict early recurrence (after less than 6 months) and create a tool for personalized treatment, Yan et al. used a combination of radiomics data (from preoperative contrast-enhanced CT, a rad-score was created using LASSO-selected radiomics features), visceral to subcutaneous fat volume ratio, and CA19–9 value to train an ML model based on logistic regression. Although other ML algorithms were not tested, this basic ML proved to have a fair predictive power (C-index = 0.712) on external validation (23).

Despite using a basic Logistic Regression model, the study achieved fair predictive power in external validation. Feature engineering (specifically, extracting radiomic features) and combining different clinical variables are often more important than the complexity of the algorithm itself.

### Predicting early liver metastasis

For the prognosis of early liver metastasis after pancreatic cancer surgery, Zhu et al. gathered different variables (BMI, fatty liver, Child–Pugh score, tumor location, N stage, differentiation, tumor emboli, nerve invasion, and adjuvant chemotherapy) from 538 patients and created and tested prognostic models based on different ML algorithms. An advanced ensemble algorithm, XG boost, provided the best performance, outperforming other similar or simpler ML methods (24). Their model was intended to improve the prediction of metastasis, facilitating personalized treatment planning.

Unlike some other prognostic areas where simple models perform best, this study found that an advanced ensemble machine learning algorithm (XGBoost) provided the best performance. It outperformed both simpler and similar ML algorithms. The model succeeded by integrating a wide range of patient and tumor characteristics rather than using only a single biomarker.

## Discussion

Machine learning provides significant benefits for the integration and assessment of vast quantities of intricate healthcare data. Compared to conventional biostatistical techniques, machine learning offers the advantages of flexibility and scalability, making it applicable to a wide range of activities, including risk stratification, diagnosis and classification, and survival prediction. The capacity of machine learning algorithms to analyze a variety of data types, such as imaging data, laboratory results, gene combinations, and pathological slides, and integrate them into forecasts for disease risk, diagnosis, prognosis, and suitable treatments is another benefit. ML has evolved from simple, basic supervised algorithms to complex, advanced algorithms, to black-box ML represented by DL.

PDAC has a dismal five-year survival rate of approximately 5%, making it one of the most lethal cancers globally (26). Currently, treating PDAC remains a clinical challenge, with the majority of patients failing to obtain survival improvements while receiving guideline-recommended therapy, which is typically due to late diagnosis or ineffective therapy. Furthermore, different treatment regimens have diverse therapeutic effects on patients, making it challenging for clinicians to choose personalized therapy programs. Using artificial intelligence technology to anticipate the prognosis of various treatment options and then choosing appropriate treatment measures based on these predictions is a promising technique.

The TNM system is the current standard for cancer staging. PDAC treatment strategies are now guided by this staging approach, which aims to identify various survival subgroups within the patient population (stages IA, IB, IIA, IIB, III, and IV). After curative resection, more than 90% of patients have metastases or cancer recurrence (27). Moreover, the overall survival rates of patients with the same TNM stage vary significantly. TNM staging’s prognostic performance reflects this diversity. For TNM staging areas, the reported c-index values for resectable and unresectable patients were 0.57 and 0.611, respectively (28–30). Therefore, it is clear that TNM staging is inaccurate in predicting outcomes, does not substantially correlate with overall survival, and is not a reliable tool for directing treatment choices (27, 31). Therefore, there is a need for improved PDAC stratification systems because of the shortcomings of traditional TNM staging. Artificial intelligence (AI) has shown encouraging results in predicting patient outcomes for different kinds of carcinomas. Its use might help to develop models with superior prediction power.

Compared to the data available for other types of cancer, such as colorectal or breast cancer, PDAC data are limited. As screening is not provided, data concerning early stage forms are scarce; survival is reduced, and successful treatment strategies are limited. Radical treatment rarely provides long-time survival; therefore, new treatment approaches and timing are necessary. Survival and recurrence predictions are vital for this purpose. Unfortunately, as stated above, the current prognosis using the TNM classification does not provide robust results, and other approaches are needed. Machine learning provides alternative variants by building different prognosis models. ML can integrate large amounts of different data (clinical, pathological, radiomics, genetic, and molecular) to create better prognosis models and guide personalized treatments. Simple ML algorithms, such as logistic regression, play a valuable role in building these models, while ensemble ML algorithms, especially Random Forests, have good predictive power. Promising predictive models have been created using a combination of DL and supervised ML or by combining two ML algorithms to improve predictive power.

To our knowledge, no other review addressing the use of ML and Advanced ML in PDAC prognosis has been published, the main reason being the novelty of the studies (less than 1 year at the time when the review was written).

## Challenges and future directions

Although the use of ML algorithms is promising, we noted that the current studies lack prospective validation, and external validation in some cases. Most studies rely on a small number of patients; therefore, their generalizability might be affected.

Considering that most studies were published in the past year, it is clear that this is just the beginning of ML algorithms in PDAC prediction. Additionally, the studies presented use different types of data, but the best type of information to use has yet to be discovered. There is no comparison between the algorithms, as the studies compare the results with the preexisting “classical” methods of prediction, not to similar research using ML methods.

Future studies are needed to confirm the reliability of these models are needed, as well as to determine the most suitable data to be analyzed and the best algorithm to be employed.

## Conclusions

ML models, from simple Logistic Regression to complex Deep Learning and Ensemble methods (such as Random Forests and XGBoost), have demonstrated a superior ability to predict survival, recurrence, and metastasis in patients with PDAC compared to conventional methods.

ML excels at integrating diverse and voluminous data (clinical, pathological, radiomics, and multi-omics/genetic) to create robust, predictive models. The development of these ML models is important for moving towards personalized therapeutic strategies, allowing clinicians to anticipate patient outcomes for different treatment options (e.g., predicting futile surgery or recurrence). Current models lack extensive external and prospective validation, which is crucial for their reliable implementation in clinical practice. Future research should focus on determining the most suitable data types and algorithms.

## Supplementary material

Supplementary Figure 1 PRISMA flow diagram of literature search [25].
