# Supplementary figures and images for: The place of advanced machine learning techniques in building pancreatic adenocarcinoma survival and recurrence prognosis models
Source: Front Oncol. 2025 Dec 17;15:1727806. doi: 10.3389/fonc.2025.1727806 (PMC12753320; doi:10.3389/fonc.2025.1727806)

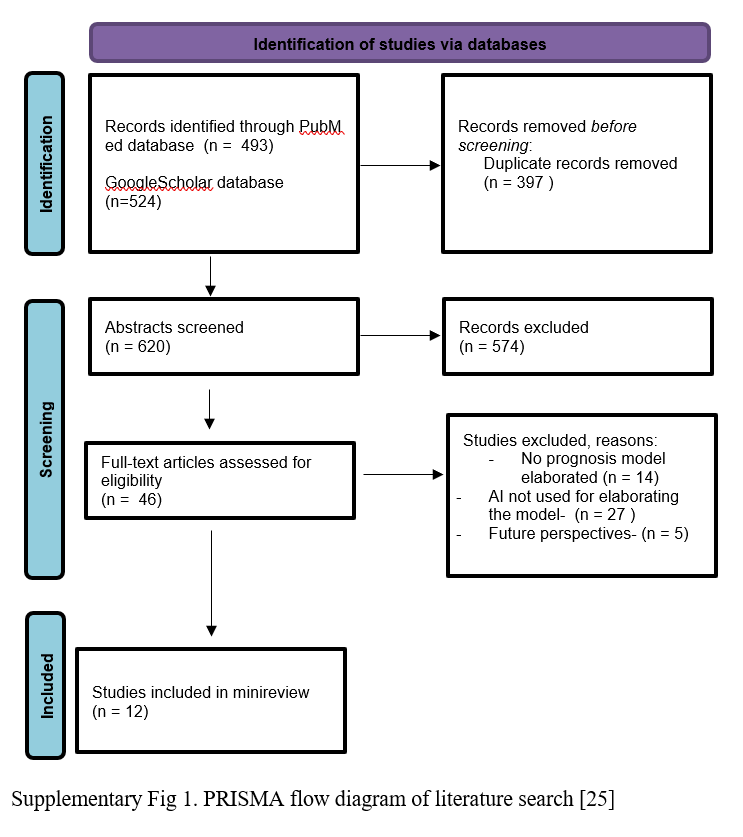

Supplement: Supplementary Figure 1 — PRISMA flow diagram of literature search [25]. [file Image1.png]
